# Analysis of von Willebrand Disease in the “Heart of Europe”

**DOI:** 10.1055/s-0042-1757635

**Published:** 2022-10-19

**Authors:** Inge Vangenechten, Petr Smejkal, Jiri Zavrelova, Ondrej Zapletal, Alexander Wild, Jan Jacques Michiels, Zwi Berneman, Jan Blatny, Angelika Batorova, Tatiana Prigancova, Miroslav Penka, Alain Gadisseur

**Affiliations:** 1 Haemostasis Unit, Antwerp University Hospital, Edegem, Belgium; 2 Medicine and Health Sciences, Haemostasis Research Unit, Antwerp University, Antwerp, Belgium; 3 Antwerp University, Antwerp, Belgium; 4 Department of Clinical Haematology, University Hospital Brno, Brno, Czech Republic; 5 Department of Laboratory Methods, Faculty of Medicine, Masaryk University, Brno, Czech Republic; 6 Department of Pediatric Haematology, University Hospital Brno, Brno, Czech Republic; 7 Department of Haematology, University F. D. Roosevelt Hospital, Banská Bystrica, Slovakia; 8 Blood Coagulation and Vascular Medicine Center, Goodheart Institute & Foundation in Nature Medicine, Rotterdam, The Netherlands; 9 Department of Haematology, Antwerp University Hospital, Edegem, Belgium; 10 National Hemophilia Center, Department of Haematology and Blood Transfusion of the Medical School of the Comenius University, Bratislava, Slovakia

**Keywords:** classification, genotype, phenotype, von Willebrand disease, von Willebrand factor

## Abstract

**Background** von Willebrand disease (VWD) is a genetic bleeding disorder caused by defects of von Willebrand factor (VWF), quantitative (type 1 and 3) or qualitative (type 2). The laboratory phenotyping is heterogenic making diagnosis difficult.

**Objectives** Complete laboratory analysis of VWD as an expansion of the previously reported cross-sectional family-based VWD study in the Czech Republic (BRNO-VWD) and Slovakia (BRA-VWD) under the name “Heart of Europe,” in order to improve the understanding of laboratory phenotype/genotype correlation.

**Patients and Methods** In total, 227 suspected VWD patients were identified from historical records. Complete laboratory analysis was established using all available assays, including VWF multimers and genetic analysis.

**Results** A total of 191 patients (from 119 families) were confirmed as having VWD. The majority was characterized as a type 1 VWD, followed by type 2. Multimeric patterns concordant with laboratory phenotypes were found in approximately 83% of all cases. A phenotype/genotype correlation was present in 84% (77% type 1, 99% type 2, and 61% type 3) of all patients. Another 45 candidate mutations (23 novel variations), not found in the initial study, could be identified (missense 75% and truncating 24%). An exon 1–3 gene deletion was identified in 14 patients where no mutation was found by direct DNA sequencing, increasing the linkage up to 92%, overall.

**Conclusion** This study provides a cross-sectional overview of the VWD population in a part of Central Europe. It is an addition to the previously published BRNO-VWD study, and provides important data to the International Society of Thrombosis and Haemostasis/European Association for Haemophilia and Allied Disorders VWD mutation database with identification of novel causal mutations.

## Introduction

von Willebrand disease (VWD) is the most common (autosomally) inherited bleeding disorder characterized by mucocutaneous bleeding, prolonged bleeding after trauma and surgery, epistaxis, and menorrhagia. 1 It is caused by genetic defects in the von Willebrand factor (VWF) gene resulting in either a quantitative deficiency (type 1 and 3) or dysfunction (type 2) of VWF. The *VWF* gene (178kb) is located on chromosome 12 (12p13.31) and consists of 52 exons spread over domains D1-D2-D'-D3-A1-A2-A3-D4-C1-C2-C3-C4-C5-C6-CK with different functional properties. 2

VWF plays an important role in primary hemostasis. During vascular injury it binds to exposed subendothelial proteins (e.g. collagen 3 ) where the adhered VWF binds to platelets through glycoprotein Ib (GPIb) alpha, 4 allowing platelet adhesion to the sites of vascular injury. VWF can also further enhance platelet aggregation through binding of GPIIb/IIIa. 5

VWF also acts as carrier for factor VIII (FVIII) in plasma, protecting it from rapid proteolytic degradation and exposing it to the site of vascular damage, thus indirectly also contributing to the plasmatic process.

This multifunctional nature of VWF contributes to the heterogeneity in clinical symptoms and bleeding risk, but also to the diagnostic challenge of VWD.

The current International Society of Thrombosis and Haemostasis-Scientific and Standardization Committee (ISTH-SSC) classification on VWD 6 recognizes three primary types, with type 1 as the most common variant, characterized by a partial quantitative deficiency of a functionally normal VWF, and type 3 VWD with a severe deficiency. Type 2 VWD is characterized by a qualitatively deficient VWF showing decreased VWF activity/VWF antigen (VWF:Ag) or factor VIII clotting factor activity (FVIII:C)/VWF:Ag. Different functional assays allow for further classification of type 2 into four subtypes: 2A, 2B, 2M, and 2N (secondary levels). 6 Beyond the ISTH classification, type 2A VWD can be further divided into four subgroups (IIA, IIC, IID, IIE, tertiary levels), based on the unique multimeric pattern that results from mechanisms that include defective multimerization, degradation of VWF, and increased proteolysis by ADAMTS-13. 7 8 9 10 11 Other type 2 VWDs are characterized by their high affinity for platelet GPIb (type 2B), or defective binding to platelets and/or subendothelial collagen (type 2M), or defective binding of VWF to FVIII leading to a mild hemophilia phenotype (type 2N). 12

A complete laboratory investigation looking at the key functions of the VWF protein is required for a complete diagnosis and classification of VWD and to establish the best treatment modality 13 : VWF:Ag, FVIII:C, VWF:ristocetin cofactor activity (VWF:RCo) and/or VWF:GPIb binding activity (VWF:GPIb), VWF collagen binding capacity (VWF:CB), VWF propeptide (VWFpp), VWF:FVIII binding capacity (VWF:FVIIIB), VWF multimeric (VWF:MM) analysis, and genetics. 2 8 The use of an algorithm of all available laboratory tests for the investigation of suspected VWD can improve its diagnosis and classification.

It is known that VWF levels (meaning the laboratory phenotypes) are influenced dramatically relative to the type of assay 14 that has been used, the percentage of coefficient of variation (%CV) of the different assays, and intra-individual variations (physical activities, stress, inflammation, medication, hormonal influences). Also, other factors can influence the VWF levels; race, age, and blood group making the diagnosis of VWD, especially type 1, difficult. 12 Therefore, the clinical aspects and personal and family history of bleeding are also required for a correct VWD diagnosis and to determine whether the inheritance is autosomal, dominant, or recessive.

Like other landmark population studies into VWD, 15 16 17 we catalogued and classified VWD patients with all available techniques including genetic analysis of the *VWF* gene to improve the understanding of the laboratory phenotype–genotype relationship in VWD. Therefore, a cross-sectional, family-based analysis of a large cohort of patients with suspected or known VWD was initiated previously focusing on the South Moravian region of the Czech Republic. This was published under the title “ *Analysis of von Willebrand Disease in the South Moravian Population (Czech Republic): Results from the BRNO-VWD Study”* 18 in 2019.

The main aim of the present study was to expand our existing patient cohort with additional samples from the Czech Republic and the neighboring Slovak Republic in order to perform laboratory phenotype–genotype analysis for complete diagnosis and classification within VWD in a geographical region of Central Europe, which we arbitrarily named “the Heart of Europe.” This article reports on the additional data gathered for a complete picture that should be combined with the already published results.

## Patients and Methods

### Study Design

The study is an extension of our previous BRNO-VWD study, 18 which reported on 166 completely characterized VWD patients, by including additional samples for a cross-sectional, family-based characterization of VWD in a large geographical region; the South Moravian area of the Czech Republic (1.9 million people) and the adjoining Slovak Republic with 5.5 million inhabitants under the arbitrary description as the “Heart of Europe.” Patients were enrolled and blood samples collected by the University Hospital Brno (Czech Republic) and the University Hospital in Bratislava and F. D. Roosevelt Hospital in Banská, Bystrica (both in Slovak Republic).

The aim of the study was to perform a laboratory phenotype–genotype analysis in order to completely diagnose and subtype VWD in each patient/family, and provide data on *VWF* mutations. The local ethical review committees of all centers approved the study protocol. Informed consent was obtained from all subjects in accordance with the Declaration of Helsinki. The study design, including data collection, patient samples, and the inclusion criteria of the participants (based on “historical” diagnosis of VWD), has been described in more detail in our previous paper. 18

### Laboratory Phenotyping of VWD

Coagulation studies were undertaken as described previously. 18 The local laboratories performed platelet function assay (PFA-100), ristocetin-induced platelet aggregation (RIPA), FVIII:C, VWF:Ag, and VWF:GPIb. The Antwerp study center defined the laboratory phenotype using additional tests: VWF:CB, VWFpp, and VWF:FVIIIB (if indicated). Where local test results were deemed discrepant and not in line with the findings of other VWF parameter results, the tests were repeated by the Antwerp study center as described before. 18

### VWF Multimeric analysis

The in-house VWF multimeric analysis was performed as previously described. 18 Additionally, the semi-automated Hydragel VW multimer assay was performed on the HYDRASYS-2 Scan system (Sebia, Lisses, France) using the HYDRAGEL 5- or 11-VW multimer kit (H5/11VWM, Sebia), according to the manufacturer's instructions, 19 20 21 22 23 24 and the use of pathological reference ranges for VWF multimer distribution. 25 The H5/11VWM was performed on all patients with VWF:GPIb/VWF:Ag and/or VWF:CB/VWF:Ag <0.90. Normally a ratio cut-off of 0.60 17 is used to distinguish between type 2 and type 1 VWD, but when taken into account the high %CV of these individual assays, a margin up to 0.90 was used for this performance.

### Genetic Analysis

Genetic analysis was performed for the identification of candidate mutations either by Sanger sequencing of *VWF* amplification products spanning exons 2–52, using oligonucleotide primers for selectivity amplification of the *VWF* gene without interference from the homologous sequences in the pseudogene (spanning exons 23–34), or by multiplex ligation-dependent probe amplification (MLPA), all as described previously. 18 The obtained sequences were aligned using the Basic Local Alignment Search Tool (blast.ncbi.nlm.nih.gov; National Library of Medicine, Bethesda, Maryland, United States), and the Coffalyser software (MRC Holland, Amsterdam, The Netherlands) was used for MLPA result analysis.

Candidate mutations were cross-referenced with the ISTH/European Association for Haemophilia and Allied Disorders (EAHAD) VWD database on *VWF* mutations ( https://grenada.lumc.nl/LOVD2/VWF/home.php?select_db=VWF ). Not previously reported gene variations were subjected to three causation prediction programs for protein comparison algorithms. 26 27 28 29 30

### VWD Classification

Classification of VWD was done according to the current ISTH-SSC VWD classification standard 6 with recognition of type 1C subtype 31 and with an additional subdivision of: type 2A into 2A/IIA, IIC, IID, and IIE, 8 32 33 34 35 type 2B into typical and atypical 2B, 36 37 38 and 2M into 2M-GPIb binding defect (2M-GPIb), 33 39 40 2M-collagen binding defect (2M-CB), 41 42 43 and 2M-unclassified (U), where the *VWF* mutation (restricted to specific domain) and the multimeric pattern were considered more important for the final VWD classification ( Table 1 ). The ISTH is in the process of putting forward a new nomenclature for the different 2M subtypes.

**Table 1:** Classification of VWD

VWD type	VWF:GPIb/VWF:Ag	VWF:CB/VWF:Ag	VWF multimers	Gene location	Other characteristics
Quantitative defect of VWF					
1	Equally reduced VWF:Ag, VWF:GPIb, VWF:CB		Normal a	Whole gene	
1C 31	Equally reduced VWF:Ag, VWF:GPIb, VWF:CB				Increased VWFpp/VWF:Ag 31
3	VWF:Ag and VWF:GPIb < 5.0 IU/dL		Absent	Whole gene	
Qualitative defect of VWF					
2A 17	<0.60	<0.60	Multimeric loss Aberrant triplet 32		
2A/IIA	<0.60	<0.60	Loss HMWM + IMWM Pronounced first sub-band	A2	
2A/IIC	<0.60	<0.60	Loss HMWM Pronounced protomer	D2	
2A/IIE	<0.60	<0.60	Loss HMWM Absence of triplet	D3	
2A/IID	<0.60	<0.60	Loss HMWM Absence of triplet Odd number of monomers	CK-terminal	
2A-U	<0.60	<0.60	Loss HMWM (IMWM) Aberrant triplet	Not A2, D2, D3, CK	
2B	<0.60	<0.60	Loss HMWM	A1	Increased LD-RIPA response
a2B	>0.60	>0.60	Normal 36 37 38	A1	Increased LD-RIPA response
2M-GPIb	<0.60	>0.60	Normal a	A1 33 39 40	
2M-CB	>0.60	<0.60	Normal	A3 41 42 43	
2M-U	<0.60	>0.60	Normal	Outside A1	
2N	>0.60	>0.60	Normal	D'-D3	FVIII:C/VWF:Ag <0,60

Abbreviations: a2B, atypical type 2B VWD; Ag, antigen; CB, collagen binding; FVIII, factor VIII; GPIb, glycoprotein Ib; HMWM, high-molecular-weight multimer; IMWM, intermediate-molecular-weight multimer; LD-RIPA, low-dose ristocetin-induced platelet aggregation; U, unclassified; VWFpp, von Willebrand propeptide; VWD, von Willebrand disease.

a VWD type where the current VWD classification allows some slight abnormalities. 6 15 44

### Statistical Analysis

Data analysis was performed using the IBM SPSS statistics software for Windows, version 27.0 (SPSS Inc., IBM Corporation, Armonk, New York, United States). Results are reported in percentage (%) of the whole study cohort.

## Results

### Patients

A total of 227 new patients belonging to 134 families were enrolled into the study. In total, 191/227 patients (in 117 families) were confirmed as having VWD; 36/227 subjects were excluded on the basis of uncertainty of the VWD diagnosis because of normal VWD levels without clinical bleeding symptoms (probably not having VWD) or borderline/low VWD levels probably due to their blood group O; in both cases an absence of a *VWF* mutation was found. Of course the blood samples in the study were not those of the historical diagnosis. Their characteristics are shown in Table 2 .

**Table 2:** Characteristics of the study population

Details study cohort	Individuals, *n* = 227 (%)
Probands Family members	134 (59.0) 93 (41.0)
Blood group (serological)	
O	50 (22.0)
Non-O	61 (26.9)
Unknown	116 (51.1)
Patients	
VWD patients—proven	191 (84.1)
UP	36 (15.9)
Family members without VWD with normal levels	25 (11.0)
Borderline/low levels due to blood group O	11 (4.8)

Abbreviations: UFM, unaffected family member; VWD, von Willebrand disease.

Note: Among 227 included cases, 134 patients were the first identified cases within a family (named probands). 191 patients were confirmed as having VWD. 36 patients (all family members) were classified as unaffected patients (UP) based on either normal VWD levels and no clinical bleeding symptoms or borderline/low VWD levels due to their blood group O, and in absence of a *VWF* mutation. The ABO blood group was determined by serological determination.

### VWD Laboratory Phenotyping

Based on the classification criteria illustrated in Table 1 , almost half of the population (88/191, 46.1%) could be classified as type 1 VWD, with type 2A VWD as the second largest group with a prevalence of 42/191 (22%): 2A/IIA (12%), 2A/IIE VWD (6.8%), and 2A-U (3.1%). Type 2B, 2M, and 2N VWD represented smaller groups with 4.7, 11.5, and 3.7%, respectively ( Table 3 ). Type 2N VWD was diagnosed due to either their heterozygous type 2N mutation combined with a “null” mutation (3/7) or double heterozygosity for a type 2N mutation (1/7), or homozygosity of a type 2N mutation (3/7). Additionally, six carriers of a type 2N mutation were identified within type 1 patients. In total, 12/23 type 3 patients were found within type 1 VWD families. For 11/23 type 3 patients, no family members were included in the study.

**Table 3:** Frequency of different VWD types

Type VWD	Frequency patients, *n* = 191 (%)
Type 1	88 (46.1)
Type 2A	42 (22)
Type 2A/IIA	23 (12.0)
Type 2A/IIC	–
Type 2A/IIE	13 (6.8)
Type 2A-U	6 (3.1)
Type 2B	9 (4.7)
Type 2M-GPIb	22 (11.5)
Type 2N	7 (3.7)
Homozygous 2N VWD mutation	3 (1.6)
Compound heterozygous 2N VWD mutation	1 (0.5)
Heterozygous 2N + null allele a	3 (1.6)
Type 3	23 (12.0), 12 within type 1 family

Abbreviations: GPIb, glycoprotein Ib; U, unclassified; VWD, von Willebrand disease.

Note: VWD patients were classified in line with the current ISTH-SSC VWD classification 6 and a supplementary sub-classification of type 2A VWD into IIA, IIC, IID, and IIE.

a All null alleles were type 1 VWD Åland mutation p.Pro812Argfs*31.

Both type 1 and type 2 had reduced VWF levels, but they could have been distinguished by their VWF activity (VWF:GPIb and/or VWF:CB) to VWF:Ag ratios with a cut-off >0.6 denoting a type 1 and <0.6 denoting a type 2. Type 2A (except 2A/IIE) and type 2B obtained reduced ratios for VWF:GPIb and VF:CB to VWF:Ag, in contrast to type 2M, which revealed only a reduced ratio for VWF:GPIb. Median VWD parameter levels for each VWD (sub-)type at time of inclusion are shown in Table 4 .

**Table 4:** Median VWD parameter levels for each VWD (sub-)type at the time of inclusion

VWD patients ( *n* = 191)	Median values [ref. range]								
	FVIII:C IU/dL (95% CI)	VWF:Ag IU/dL (95% CI)	VWF:GPIb IU/dL (95% CI)	VWF:CB IU/dL (95% CI)	VVWFpp IU/dL (95% CI)	FVIII:C/VWF:Ag	VWF:GPIb/VWF:Ag	VWF:CB/VWF:Ag	VWFpp/VWF:Ag
	[60–140]	[60–160]	[60–150]	[60–150]	[60–140]	[>0.60]	[>0.60]	[>0.60]	[<1.50]
Type1 *(n* = 88)	71.0 (68.1–82.6)	40.0 (40.8–49.1)	38.0 (37.6–45.8)	39.0 (38.1–48.0)	57.0 (54.3–66.6)	1.76 (1.65–1.97)	0.96 (0.89–0.98)	0.97 (0.90–0.99)	1.33 (1.26–1.71)
Type 2 ( *n* = 80)	47.0 (44.2–56.8)	47.0 (41.6–53.0)	14.0 (16.5–26.7)	18.0 (30.9–31.9)	93.0 (87.9–112.4)	1.2 (1.1–1.4)	0.39 (0.42–0.57)	0.60 (0.52–0.69)	2.23 (2.20–2.71)
Type 2A ( *n* = 42)	48.0 (43.4–60.3)	43.0 (37.4–52.5)	12.0 (11.0–16.4)	10.0 (10.9–18.4)	98.0 (90.4–132.3)	1.16 (1.15–1.44)	0.28 (0.31–0.50)	0.33 (0.31–0.52)	2.44 (2.33–3.03)
Type 2A/IIA ( *n* = 23)	48.0 (42.2–56.1)	50.0 (41.3–57.6)	12.0 (8.5–16.2)	6.0 (4.6–12.6)	101.0 (91.4–132.2)	1.04 (0.94–1.15)	0.22 (0.19–0.35)	0.13 (0.11–0.29)	2.23 (2.05–2.51)
Type 2A/IIE ( *n* = 13)	39.0 (30.6–63.9)	24.0 (15.6–47.3)	16.0 (12.7–23.3)	18.0 (13.2–23.2)	79.0 (59.4–119.8)	1.79 (1.46–1.98)	0.72 (0.55–0.87)	0.72 (0.55–0.96)	3.29 (2.64–4.43)
Type 2A-U ( *n* = 6)	51.0 (24.5–114.1)	53.0 (26.2–83.8)	7.0 (3.7–16.6)	22.0 (13.9–41.8)	134.0 (31.7–269.1)	1.30 (0.76–1.88)	0.16 (0.00–0.57)	0.53 (0.47–0.57)	2.62 (1.32–3.48)
Type 2B ( *n* = 9)	49.0 (30.7–79.6)	47.0 (38.2–68.2)	14.0 (7.9–22.1)	18.0 (10.1–26.5)	121.0 (97.3–137.4)	0.88 (0.74–1.34)	0.20 (0.12–0.60)	0.33 (0.18–0.61)	2.55 (1.87–2.89)
Type 2M-PIb ( *n* = 22)	55.5 (42.0–68.6)	36.0 (30.0–55.2)	16.0 (14.3–31.2)	28.0 (27.6–52.5)	86.5 (65–92.7)	1.51 (1.29–2.01)	0.53 (0.44–0.69)	0.95 (0.85–1.03)	1.98 (1.88–3.03)
Type 2N ( *n* = 7)	20.5 (15.0–28.0)	62.5 (39.6–89.5)	72.0 (33.0–115.0)	68.0 (39.1–94.3)	73.0 (57.0–85.4)	0.33 (0.29–0.40)	1.05 (0.84–1.34)	1.04 (0.83–1.24)	1.08 (0.82–1.59)

Abbreviations: Ag, antigen; CB, collagen binding; CI, confidence interval; FVIII, factor VIII; GPIb, glycoprotein Ib; pp, propeptide; n.c., not calculated; VWD, von Willebrand disease; VWF, von Willebrand factor.

Note: Although type 1 and 2 had reduced VWF levels, they could be distinguished by their VWF activity (VWF:GPIb and/or VWF:CB) to VWF:Ag ratios with a cut off >0.6 denoting a type 1 and <0.6 denoting type 2. Type 2A (except 2A/IIE) and 2B obtained reduced ratios for VWF:GPIb and VWF:CB to VWF:Ag in contrast to type 2M, which had only a reduced ratio for VWF:GPIb/VWF:Ag.

Distinction between type 2A and 2B was done by RIPA, which was done locally, but unfortunately, LD-RIPA was performed for only 6/9 type 2B patients, with 3/6 showing a pathognomonically increased LD-RIPA response (0.6 mg/mL) (normal range < 20%). Despite a normal LD-RIPA, 3/6 patients were ultimately classified as type 2B based on an aberrant VWF multimer pattern and the presence of a *VWF* mutation associated with the type 2B VWD pattern.

### VWF Multimeric Analysis

**Fig. 1:**
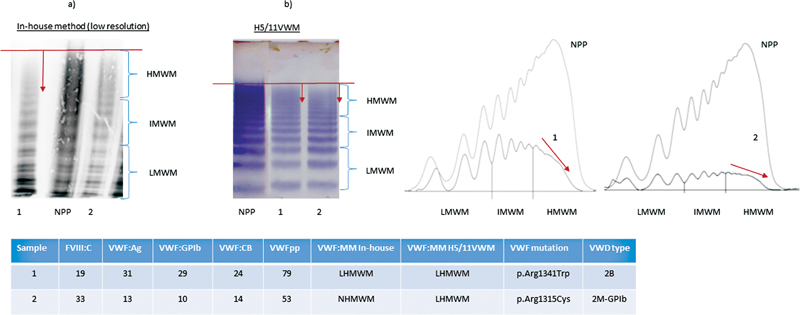
**Results of von Willebrand factor multimers, in-house and H5/11VWM, with discordant VWF multimeric pattern compared with the proposed laboratory phenotype results.** Note: Patients 1 and 2 had a laboratory phenotype consistent with type 1. Patients 1 had a loss of HMWM with both methods. Patient 2 revealed a loss of HMWM using the H5/11VWM. Normal multimers were observed using the in-house method. Both patients were ultimately classified as type 2 VWD; patient 1 as 2B and patient 2 as 2M VWD based on the aberrant multimeric patterns and the presence of a corresponding VWF mutation. Ag, antigen; CB, collagen binding; FVIII, factor VIII; GPIb, glycoprotein Ib; HMWM, high-molecular-weight multimer; IMWM, intermediate-molecular-weight multimer; LMWM, low-molecular-weight multimer; MM, multimers; NPP, normal plasma pool; VWD, von Willebrand disease; VWF, von Willebrand factor; VWFpp, von Willebrand factor propeptide.

All 191 patients underwent VWF multimeric analysis using the in-house method. For 121 patients, taking into account the 0.90 cut-off for the VWF:GPIb and/or VWF:CB to VWF:Ag ratios, also the semi-automated Hydragel VW multimeric (H5/11VWM) method was performed. Results of both methods, in-house and H5/11VWM, revealed a concordant VWF multimeric pattern in line with the proposed laboratory phenotype (VWF:Ag, VWF:GPIb, VWF:CB, VWFpp, and FVIII:C) in, respectively, 84 and 82% of all cases, and a discordant multimeric pattern compared with the proposed laboratory phenotype in, respectively, 16 and 18% of all cases ( Fig. 1 ).

A loss of high-molecular-weight multimer (HMWM) in type 1 laboratory phenotype cases was observed in 12% when measured with the in-house method and in 19% with the H5/11VWM. These patients were ultimately classified as type 2 VWD (type 2A/IIE, 2B, and 2M) based on the aberrant multimeric patterns and the presence of a corresponding *VWF* mutation.

Conversely, a normal multimeric pattern with a type 2 laboratory phenotype was observed by the in-house method and H5/11VWM in respectively 28 and 17% of all cases. Overall, the laboratory phenotype of type 2 with normal multimeric patterns was mainly seen in type 2M-GPIb patients. These patients historically are not expected to have multimeric losses, although the current VWD classification allows some slight abnormalities. 6 15 44 Remarkably, the H5/11VWM observed approximately 50% more HMWM losses in type 2M-GPIb patients than the in-house method, a phenomenon well described in our publication on the Hydragel VW multimer assay. 25

### Genetic Analysis

**Fig. 2:**
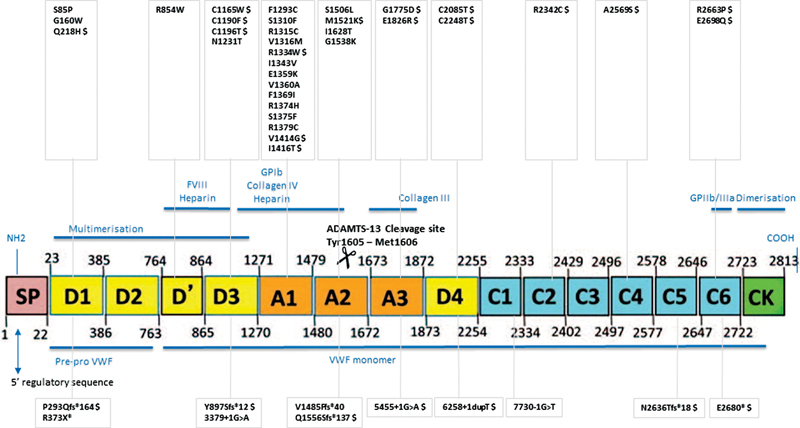
**Location of sequence variation identified in this von Willebrand disease cohort which was not observed in the initial BRNO-VWD cohort (*n* = 45).** Missense mutations are present on top ( *n* = 34), and truncating variations ( *n* = 11) are illustrated at the bottom. Novel gene variations ( *n* = 23) are marked with $.

DNA sequencing identified at least one causal mutation in 161/191 cases (84%), while for 30/191 (22/88 type 1 and 8/23 type 3) no genetic confirmation for the diagnosis of VWD was obtained. A compound heterozygous variation could be found in 27/161 patients. In comparison with the previously reported BRNO-VWD study results, 18 another 45 different candidate mutations (34 missense mutations and 11 truncating variations) were identified ( Fig. 2 ). Twenty-three of them (16 missense and 7 truncating) were not previously reported gene variations (in 32 patients), and were observed in VWD type 1 ( *n* = 19); type 2M-GPIb ( *n* = 6), type 3 ( *n* = 4), type 2A/IIE ( *n* = 2); and type 2A/IIA ( *n* = 1). The results of the “new” missense mutations after subjection to three causation prediction programs for protein comparison algorithms 26 27 28 29 30 are reported in Table 5 . In total, 8/16 “new” variants were considered “deleterious” by all three programs, and 5/16 as “benign.” The prediction programs gave conflicting results for three variants, which makes these results difficult to interpret, but since they were not found in conjunction with another “causal” mutation, the probability that they do cause a VWF defect is high.

**Table 5:** Missense mutations which are not previously reported in the ISTH/EAHAD database on *VWF* mutation and subjected to causation prediction programs for protein comparison algorithms

domain	HGVSp	HGVSc	Frequency	Type VWD	DEOGEN2 comparison	Poly-Phen2 comparison	Mutationtaster comparison	gnomAD MAF%
D1	p.Gln218His	c.654G > T	1	1	Benign	Benign	Polymorphism	UNK
D3	p.Cys1165Trp	c.3495C > G	1	1	Deleterious	Probably damaging	Disease causing	UNK
	p.Cys1190Phe	c.3569G > T	1	2A/IIE	Deleterious	Probably damaging	Disease causing	UNK
	p.Cys1196Tyr	c.3585G > A	1	2A/IIE	Deleterious	Probably damaging	Disease causing	UNK
A1	p.Arg1334Trp	c.4000C > T	2	2M-GPIb	Deleterious	Probably damaging	Disease causing	NFE: 0.0055
	p.Val1414Glu	c.2441T > A	2	2M-GPIb	Deleterious	Probably damaging	Disease causing	UNK
	**p.Ile1416Thr**	**c.4247T** **>** **C**	2	2M-GPIb	Benign	Probably damaging	Disease causing	NFE: 0.0009
A2	**p.Met1521Lys**	**c.4562T** **>** **A**	1	2A/IIA	Benign	Benign	Disease causing	UNK
A3	p.Gly1775Asp	c.5324G > A	1	1 a	Benign	Benign	Polymorphism	UNK
	p.Gly1826Arg	c.5476G > A	2	1	Deleterious	Probably damaging	Disease causing	UNK
D4	p.Cys2085Tyr	c.6254G > A	2	1	Deleterious	Probably damaging	Disease causing	UNK
	p.Cys2248Tyr	c.6737G > A	1	1	Deleterious	Probably damaging	Disease causing	UNK
	p.Arg2342Cys	c.7024C > T	1	1	Benign	Benign	Polymorphism	NFE: 0.0018
C4	p.Ala2569Ser	c.7706G > T	1	1 a	Benign	Benign	Polymorphism	UNK
C6	p.Arg2663Pro	c.7988G > C	1	1	Benign	Benign	Polymorphism	ALL: 0.1429
	**p.Glu2698Gly**	**c.8093A** **>** **G**	1	1	Deleterious	Benign	Polymorphism	UNK

Abbreviations: ALL, all-round population; EAHAD, European Association for Haemophilia and Allied Disorders; ISTH, International Society of Thrombosis and Haemostasis; MAF, minor allele frequency; NFE, not finished European population; UNK, variant not found in other populations; VWD, von Willebrand disease.

Note: In bold, novel mutation with disagreement in prediction score. The gnomAD MAF frequency (%) indicates whether a variant has been found in a healthy population with a significant MAF score. In total, 8/16 variations were predicted to be deleterious, 5/16 as benign, and 3/16 were doubtful.

a Compound heterozygous with another mutation.

**Fig. 3:**
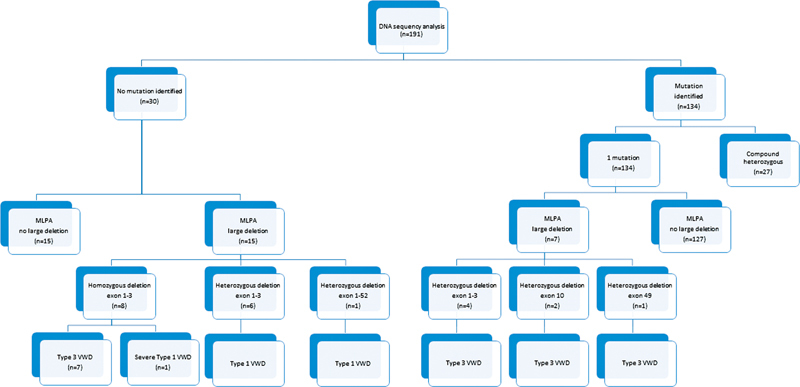


For all 191 VWD patients, the MLPA was performed revealing a large deletion in 22/191 patients ( Fig. 3 ); 15/22 in type 3 and 7/22 in type 1. A heterozygous deletion of exons 1–3 ( *n* = 10), exon 10 ( *n* = 2), exon 49 ( *n* = 1), and exons 1–52 ( *n* = 1), and a homozygous deletion of exons 1–3 ( *n* = 8) were found.

A total of seven patients with a heterozygous deletion (exons 1–3 [ *n* = 4], exon 10 [ *n* = 2], or exon 49 [ *n* = 1]) were compounded by a gene variation already identified by DNA sequencing, resulting in type 3 VWD.

For 15 patients with a large deletion (homozygous exons 1–3 [ *n* = 8], heterozygous exons 1–3 [ *n* = 6], and heterozygous exons 1–52 [ *n* = 1]), no “extra” mutation was found by direct DNA sequencing, which increased the linkage from approximately 84% up to approximately 92% over all types.

### Laboratory Phenotype/Genotype correlation

The proposed laboratory phenotypes (based on VWF:Ag, VWF:GPIb, VWF:CB, VWFpp, and FVIII:C) were correlated with the multimeric and genetic analysis results ( Supplementary Table S1 ). In total, 89% of the identified gene variations confirmed the proposed laboratory phenotypic and multimeric results. Eight amino acid substitutions (in 36 patients) were observed in different laboratory phenotypes ( Table 6 ).

**Table 6:** Amino acid substitutions occurring in different von Willebrand disease phenotypes

HGVSp	ISTH/EAHAD class	Laboratory phenotype	Frequency	Final VWD classification	Remarks
**D1 domain**					
p.Pro293Glnfs*164	–	2M	1/1	1	Final classification based on NHMWM and D1 domain mutation
**D3 domain**					
p.Trp1144Gly	1 >> 2A	1	6/8	2A/IIE	Final classification based on LHMWM and D3 domain mutation
		2A	1/8	2A/IIE	–
		3	1/8	3	Compound heterozygous with large gene deletion
p.Tyr1146Cys	1 >> 2A	1	1/2	2A/IIE	Final classification based on NHMWM and D3 domain mutation (within 1 family)
		2A	1/2	2A/IIE	
**p.Cys1196Tyr**	–	1	1/1	2A/IIE	Final classification based on LHMWM and D3 domain mutation
**A1 domain**					
p.Arg1315Cys	1 (2A), 2M, U	1	5/9	2M-GPIb	Final classification based on A1 domain mutation, NHMWM with in-house and LHMWM with H5/11HVWM
		2M-GPIb	4/9	2M-GPIb	
**p.Arg1334Trp**	–	1	1/2	2M-GPIb	Final classification based on NHMWM and A1 domain mutation (within 1 family)
		2M-GPIb	1/2	2M-GPIb	
**p.Val1414Glu**	–	1	1/2	2M-GPIb	Final classification based on A1 domain mutation, NHMWM with in-house and LHMWM with H5/11HVWM (within 1 family)
		2M-GPIb	1/2	2M-GPIb	
**A2 domain**					
p.Gly1579Arg	2A	1	1/11	2A/IIA	Patient with normal multimer pattern. Final classification solely based on A2 domain mutation
		2A/IIA	10/11	2A/IIA	–

Abbreviations: LHMWM, loss of high-molecular-weight multimers; NHMWM, normal high-molecular-weight mulitmers; H5/11VWM, Hydragel VW multimeric method.

Notes: In bold, mutation not previously described in the ISTH/EAHAD database.

The already reported variations in D3, p.Trp1144Gly/c.3430T > C and p.Tyr1146Cys/c.3437A > G, were found in patients with phenotypic laboratory results consistent with differing types of VWD: p.Trp1144Gly/c.3430T > C in type 1 (6/8), type 3 (1/8), and type 2A/IIE (1/8) VWD; p.Tyr1146Cys/c.3437A > G in type 1 (1/2) and 2A/IIE (1/2). In addition, 9/10 patients were ultimately classified as type 2A/IIE based on their corresponding VWF type 2A/IIE multimer pattern and the location of the mutation in the D3 domain of the VWF gene. One patient was classified as type 3 VWD because he was found to be compound heterozygous for p.Trp1144Gly/c.3430T > C and truncating variation.

Nine patients carried the p.Arg1315Cys/c.3943C > T mutation and were finally classified as type 2M-GPIb based on their normal VWF multimeric pattern (in-house method) and the A1 domain mutation. For 4/9 patients this was in line with their laboratory phenotype, but for 5/9 patients a type 1 laboratory phenotype was observed.

Although the p.Gly1579Arg/c.4735G > A mutation is generally found in type 2A/IIA patients, a type 1 laboratory phenotype was observed in one patient. This patient showed a normal multimeric pattern but was finally classified as type 2A/IIA solely based on his A2 domain mutation.

Four novel substitutions were found in laboratory phenotype type 1 and type 2M-GPIb VWD: one patient with p.Pro293Glnfs*164/c.878delC had a type 2M phenotype but was ultimately classified as type 1 VWD due the mutation located in D1, rather than in A1. Although one patient with p.Cys1196Tyr/c.3585G > A had a type 1 phenotype, he was classified as type 2A/IIE based on an aberrant multimeric pattern consistent with type 2A/IIE in conjunction with a mutation in D3.

Mutations p.Arg1334Trp/c.4000C > T and p.Val1414Glu/c.2441T > A were found in patients with a laboratory phenotype consistent with type 1 and type 2M, respectively. Based on their aberrant multimeric pattern and the location of the mutation within the A1-domain, they were finally classified as 2M-GPIb.

For one patient, a homozygous exon 1–3 deletion was observed, which had a laboratory phenotype mimicking a type 1 VWD with the presence of VWF multimers, but where a relative loss of HMWM was seen. Based on these findings, this patient was classified as a severe type 1 VWD.

## Discussion

In 2018, we published the results of our cross-sectional, family-based, VWD study, under the title “ *Analysis of von Willebrand Disease in the South Moravian Population (Czech Republic): Results from the BRNO-VWD study.”* 18 Now, we report on the results of additional samples from the Czech and Slovak Republics in an extension of our previous study. The study was focused on improving the knowledge of VWD laboratory phenotype–genotype relationship in a particular geographical region, termed “the Heart of Europe.” This entailed a complete laboratory investigation of a cohort of 227 VWD patients, included based on “historical” results, with the study work done on new purposely collected blood samples. The study was carried out by the University Hospital Brno (Czech Republic), University Hospital in Bratislava, and F. D. Roosevelt Hospital in Banská, Bystrica (both in Slovak Republic), and the Antwerp University Hospital (Belgium).

Our study catalogued and classified VWD patients with all available techniques. According to the current ISTH-SSC classification, 6 patients were classified into type 1, 2A, 2B, 2M, 2N, or 3, with a subdivision of type 2A into 2A/IIA, IIC, IID, and IIE, 8 32 33 34 35 type 2B into typical and atypical 2B, 36 37 38 and 2M into 2M-GPIb, 33 39 40 2M-CB, 41 42 43 and U.

A total of 191 patients were confirmed as having VWD and around half of them could be classified as type 1 VWD, type 2A (2A/IIA and 2A/IIE) as the second largest group, and with type 2B (typical), 2M-GPIb, and 2N as less frequent types. This distribution has been reported before 15 16 17 45 and is in line with our own previous reported findings. 18 In addition, 50% of type 3 patients were found within type 1 VWD families. For the other 50%, no family history or results were available.

Concordant results between the VWF multimer pattern, restricted to the current ISTH-SSC classification, 6 and the observed laboratory phenotype (FVIII:C, VWF:Ag, VWF:GPIb, VWF:CB, and VWFpp) were found in approximately 83% of the entire study population. Around 17% of all cases showed a multimeric pattern discrepant with the laboratory phenotype, but the ultimate classification was validated by the corresponding *VWF* mutations.

Although genetic analysis is not used in routine practice to diagnose VWD, it was, in conjunction with the ISTH/EAHAD database on *VWF* mutations, used as a final arbiter to classify VWD. Although the database contains most known *VWF* mutations, the VWD classification depends on the laboratory panel and sometimes the same mutation is ascribed to multiple VWD types. As a consequence, there is no gold standard in VWD classification.

Using DNA sequencing, at least one underlying causative mutation was found in 84% of all patients. We were able to identify another 45 candidate mutations which were not previously observed in the initial BRNO-VWD cohort. 15 The Åland mutation (p.Pro812Argfs*31, type1) continues to be the most frequent mutation (15%) in all VWD cases, which has also been reported in other European countries. 15 16 17

Twenty-three novel *VWF* gene variations were identified with 19 confirming the laboratory phenotype and multimeric pattern. MLPA identified large deletions of exon 1–3 46 in 15 VWD patients (7 type 3 and 8 type 1), where no underlying mutation had been found by direct DNA sequencing, accounting for a linkage of 92% of all VWD patients, overall, and 83% of all type 1 patients, a figure which has also been reported in other population studies. 15 17 47 48 49 50 But it has to be noted that the rate of genetic variants observed in VWD type 1 patients can be attributed to their inclusion criteria. Subjects with VWF levels <30 IU/dL are more likely to have a VWF variant than those with levels ranging between 30 and 50 IU/dL. Still, there will be a number of VWD patients, especially type 1, who do not have an associated *VWF* mutation.

It is already known that a number of loci outside the *VWF* gene have been shown to affect the VWF levels. 51 52 Therefore, patients without an identified causal mutation could go forward for investigation of other genes outside the *VWF* locus which implicate in alternating VWF levels.

In general, all results observed for the samples included by the Slovak Republic were very similar to those included by the Czech Republic, unsurprisingly given that both Republics are neighboring states and they formed parts of Czechoslovakia until 1993, and historically find their ancestry in Great Moravia. Therefore, all included samples by both Republics can be considered as a single population cohort in future study projects or comparisons with other regions.

This cross-sectional VWD study termed the “Heart of Europe” demonstrates that the use of a basic VWF assay panel (FVIII:C, VWF:Ag, VWF:GPIb, VWF:CB, and VWFpp) is not enough for an accurate VWD classification, since it is dramatically influenced by several (pre-)analytical and inter-individual variations. 12 14 It shows that an extensive panel of all VWD assays with all available techniques, including VWF multimer analysis and a full genetic analysis (DNA sequencing and MLPA), is required for a complete VWD diagnosis and classification, as it was done in other VWD population studies. 15 16 17 It provides unique information on the laboratory phenotype–genotype relationship in VWD, including the pitfalls in diagnosis of the disease, but adds the data of a large geographical region into the ISTH/EAHAD database on *VWF* mutations. This study and several other population studies 15 16 17 in VWD invite comparisons between the different mutations identified in different regions. They could be used to follow population migration patterns which can be valuable materials for future studies into VWD.

## Notes

**What Is Known about This Topic?**

- The diagnosis and classification of von Willebrand is not standardized.
- The laboratory phenotype is highly heterogeneous due to assay variations and intra-individual variations, which makes the diagnosis of von Willebrand disease difficult.

**What Does This Paper Add?**

- We provide unique information on a large VWD cohort in central Europe in order to understand the phenotype–genotype relationship in VWD.
- The study illustrates current problems in VWD classification/subtyping.
