# Supplementary material for: Analysis of von Willebrand Disease in the “Heart of Europe”
Source: TH Open. 2022 Oct 19;6(4):e335–46. doi: 10.1055/s-0042-1757635 (PMC9581583; doi:10.1055/s-0042-1757635)
Supplement: Supplementary file 1 — Supplementary Material [file 10-1055-s-0042-1757635-s22060029.pdf]

Supplementary Table S1 Laboratory phenotype–genotype correlation

FN	UPN	FVIII	VWF: Ag	VWF: GPIb	VWF: CB	VWFpp	FVIII:C/ VWF:Ag	VWF: GPIb/ VWF:Ag	VWF:CB/ VWF:Ag	VWFpp/ VWF:Ag	Laboratory phenotype	VWF:MM, in-house	VWF:MM, Hydragel	HGVSp	HGVSc	Genotype	MLPA large deletion	Genotype	VWD type
1	1	59	75	57	71	38	0.79	0.76	0.95	0.51	1	NHMWM	NHMWM	p.Pro812Argfs*31	c.2435delC	het	Normal		1
2	2	47	78	7	43	168	0.60	0.09	0.55	2.15	2A/B	AHMWM + LIMWM	LHMWM	p.Arg1374His	c.4121G > A	het	Normal		2A-U
	3	20	26	6	15	68	0.77	0.23	0.58	2.62	2A/B	AHMWM + LIMWM	LHMWM	p.Arg1374His	c.4121G > A	het	Normal		2A-U
	4	168	110	19	53	412	1.53	0.17	0.48	3.75	2A/B	AHMWM + LIMWM	LHMWM	p.Arg1374His	c.4121G > A	het	Normal		2A-U
3	5	50	64	59	63	106	0.78	0.92	0.99	1.66	1	NHMWM	n.d.	p.Pro1266Leu<br>p.val1279Ile	c.3797C > T<br>c.3835G > A	het<br>het	Normal		1
5	6	3	0	15	0	0		0.00		0.00	3	absent	n.d.	p.Pro812Argfs*31<br>p.Glu2680* §	c.2435delC<br>c.8038G > T §	het<br>het	Exon 49	het	3
	7	65	29	45	30	53	2.24	1.55	1.04	1.83	1	NHMWM	n.d.	p.Pro812Argfs*31	c.2435delC	het	Normal		1
	8	93	75	67	83	94	1.24	0.89	1.11	1.25	1	NHMWM	NHMWM	p.Glu2680* §	c.8038G > T §	het	Normal		1
6	9	2	0	0	0	0		0.00		0.00	3	absent	n.d.	No causative mutation found			Exon 1–3	hom	3
7	10	57	47	36	53	88	1.21	0.77	1.14	1.87	1	NHMWM	NHMWM	p.Arg1334Trp §	c.4000C > T §	het	Normal		2M-GPIb
	11	23	55	18	37	62	0.42	0.33	0.67	1.13	2M-GPIb	NHMWM	NHMWM	p.Arg1334Trp §	c.4000C > T §	het	Normal		2M-GPIb
8	12	73	64	65	88	54	1.14	1.02	1.38	0.84	1	NHMWM	n.d.	p.Pro812Argfs*31	c.2435delC	het	Normal		1
9	13	80	58	64	55	97	1.38	1.10	0.95	1.67	1	NHMWM	n.d.	p.Tyr1584Cys	c.4751A > G	het	Normal		1
10	14	4	0	0	0	0		0.00		0.00	3	absent	n.d.	p.Arg1853*	c.5557C > T	het	Exon 1–3	het	3
12	15	28	27	12	15	26	1.04	0.44	0.56	0.96	2A/B	LHMWM	LHMWM	p.Met1521Lys §	c.4562T > A §	het	Normal		2A/IIA
4	16	52	64	12	6	136	0.81	0.19	0.09	2.13	2A	AHMWM + LIMWM	AHMWM + LIMWM	p.Gly1579Arg	c.4735G > A	het	Normal		2A/IIA
	17	63	90	17	6	199	0.70	0.19	0.07	2.21	2A	AHMWM + LIMWM	AHMWM + LIMWM	p.Gly1579Arg	c.4735G > A	het	Normal		2A/IIA
	18	87	62	15	7	141	1.40	0.24	0.11	2.27	2A	AHMWM + LIMWM	AHMWM + LIMWM	p.Gly1579Arg	c.4735G > A	het	Normal		2A/IIA
	19	55	49	12	6	101	1.12	0.24	0.12	2.06	2A	AHMWM + LIMWM	AHMWM + LIMWM	p.Gly1579Arg	c.4735G > A	het	Normal		2A/IIA
	20	70	78	15	5	187	0.90	0.19	0.06	2.40	2A	AHMWM + LIMWM	AHMWM + LIMWM	p.Gly1579Arg	c.4735G > A	het	Normal		2A/IIA
	21	51	65	14	5	179	0.78	0.22	0.08	2.75	2A	AHMWM + LIMWM	AHMWM + LIMWM	p.Gly1579Arg	c.4735G > A	het	Normal		2A/IIA
	22	62	59	12	6	93	1.05	0.20	0.10	1.58	2A	AHMWM + LIMWM	AHMWM + LIMWM	p.Gly1579Arg	c.4735G > A	het	Normal		2A/IIA
	23	61	59	11	10	183	1.03	0.19	0.17	3.10	2A	AHMWM + LIMWM	AHMWM + LIMWM	p.Gly1579Arg	c.4735G > A	het	Normal		2A/IIA
	24	47	43	14	6	96	1.09	0.33	0.13	2.23	2A	AHMWM + LIMWM	AHMWM + LIMWM	p.Gly1579Arg	c.4735G > A	het	Normal		2A/IIA
23	25	164	93	67	90	67	1.76	0.72	0.97	0.72	1	NHMWM	n.d.	p.Pro812Argfs*31	c.2435delC	het	Normal		1
29	26	49	46	14	5	154	1.07	0.30	0.11	3.35	2A	LHMWM	LHMWM	p.Arg854Gln<br>p.Arg1308Cys	c.2561G > A<br>c.3922C > T	het het	Normal		2B
53	27	54	25	24	18	87	2.16	0.96	0.72	3.48	1	LHMWM	LHMWM	p.Trp1144Gly	c.3430T > G	het	Normal		2A/IIE
66	28	28	22	9	24	112	1.27	0.41	1.09	5.09	2M-GPIb	NHMWM	NHMWM	p.Lys1408fs	c.4222–4224delAAG	het	Normal		2M-GPIb
	29	78	42	16	32	136	1.86	0.38	0.76	3.24	2M-GPIb	NHMWM	NHMWM	p.Lys1408fs	c.4222–4224delAAG	het	Normal		2M-GPIb
97	30	36	43	30	35	113	0.84	0.70	0.81	2.63	1	LHMWM	NHMWM	p.Arg1341Trp	c.4021C > T	het	Normal		2B
	31	46	28	17	18	92	1.64	0.61	0.64	3.29	1	LHMWM	LHMWM	p.Arg1341Trp	c.4021C > T	het	Normal		2B
	32	19	31	29	24	79	0.61	0.94	0.77	2.55	1	LHMWM	LHMWM	p.Arg1341Trp	c.4021C > T	het	Normal		2B
98	33	74	57	32	53	97	1.30	0.56	0.93	1.70	2M-GPIb	NHMWM	NHMWM	p.Phe1293Cys	c.3878T > G	het	Normal		2M-GPIb
99	34	29	40	32	30	56	0.73	0.80	0.75	1.40	1	NHMWM	NHMWM	No causative mutation found			Normal		1
100	35	85	35	32	32	47	2.43	0.91	0.91	1.34	1	NHMWM	n.d.	p.Cys2248Tyr §	c.6737G > A §	het	Normal		1

(Continued)

 © 2022. The Author(s).

Supplementary Table S1 (*Continued*)

FN	UPN	FVIII	VWF: Ag	VWF: GPIb	VWF: CB	VWFpp	FVIII:C/ VWF:Ag	VWF: GPIb/ VWF:Ag	VWF:CB/ VWF:Ag	VWFpp/ VWF:Ag	Laboratory phenotype	VWF:MM, in-house	VWF:MM, Hydragel	HGVSp	HGVSc	Genotype	MLPA large deletion	Genotype	VWD type
101	**36**	65	60	32	39	87	1.08	0.53	0.65	1.45	2M-GPIb	NHMWM	LHMWM		c.5455 + 1G > A §	het	Normal		**1**
102	**37**	54	34	24	20	13	1.59	0.71	0.59	0.38	2M-CB	NHMWM	NHMWM	p.Arg2663Pro §	c.7988G > C §	het	Normal		**1**
103	**38**	70	48	44	29	62	1.46	0.92	0.60	1.29	1	NHMWM	NHMWM	p.Val1485Phefs*40	c.4453delG	het	Normal		**1**
	**39**	105	66	45	41	72	1.59	0.68	0.62	1.09	1	NHMWM	NHMWM	p.Val1485PhefsX40	c.4453delG	het	Normal		**1**
	**40**	120	57	61	42	53	2.11	1.07	0.74	0.93	1	NHMWM	NHMWM	p.Val1485PhefsX40	c.4453delG	het	Normal		**1**
104	**41**	48	69	3	6	155	0.70	0.04	0.09	2.25	2A	AHMWM + LIMWM	AHMWM + LIMWM	p.Gly1609Arg	c.4825G > A	het	Normal		**2A/IIA**
105	**42**	88	57	50	34	73	1.54	0.88	0.60	1.28	1	NHMWM	NHMWM	No causative mutation found			Normal		**1**
	**43**	93	82	79	68	77	1.13	0.96	0.83	0.94	1	NHMWM	NHMWM	No causative mutation found			Normal		**1**
106	**44**	36	18	7	10	69	2.00	0.39	0.56	3.83	2A	LHMWM	LHMWM	p.Cys1130Gly	c.3388T > G	het	Normal		**2A/IIE**
	**45**	112	59	17	21	191	1.90	0.29	0.36	3.24	2A	LHMWM	LHMWM	p.Cys1130Gly	c.3388T > G	het	Normal		**2A/IIE**
107	**46**	42	44	36	48	106	0.95	0.82	1.09	2.41	1	NHMWM	NHMWM	p.Arg924Gln	c.2771G > A	het	Normal		**1**
108	**47**	71	32	30	32	41	2.22	0.94	1.00	1.28	1	NHMWM	n.d.	p.Asn166Ile	c.497A > T	het	Normal		**1**
109	**48**	48	33	32	38	58	1.45	0.97	1.15	1.76	1	NHMWM	n.d.	p.Gly1826Arg §	c.5476G > A §	het	Normal		**1**
	**49**	54	41	27	40	79	1.32	0.66	0.98	1.93	1	NHMWM	NHMWM	p.Gly1826Arg §	c.5476G > A §	het	Normal		**1**
110	**50**	76	63	14	5	136	1.21	0.22	0.08	2.16	2A	AHMWM + LIMWM	AHMWM + LIMWM	p.Gly1579Arg	c.4735G > A	het	Normal		**2A/IIA**
111	**51**	3	6	5	6	1	0.50	0.83	1.00	0.17	1	NHMWM	NHMWM	p.Pro812Argfs*31	c.2435delC	het	Normal		**1**
112	**52**	54	19	5	13	39	2.84	0.26	0.68	2.05	2M-GPIb	NHMWM	LHMWM	p.Arg854Gln<br>p.Arg1315Cys	c.2561G > A<br>c.3943C > T	het het	Normal		**2M-GPIb**
	**53**	64	24	4	18	58	2.67	0.17	0.75	2.42	2M-GPIb	NHMWM	LHMWM	p.Arg854Gln<br>p.Arg1315Cys	c.2561G > A<br>c.3943C > T	het het	Normal		**2M-GPIb**
113	**54**	50	24	23	20	32	2.08	0.96	0.83	1.33	1	NHMWM	NHMWM	p.Pro812Argfs*31	c.2435delC	het	Normal		**1**
114	**55**	61	46	39	34	92	1.33	0.85	0.74	2.00	1	NHMWM	NHMWM	p.Tyr1584Cys	c.4751A > G	het	Normal		**1**
115	**56**	28	82	85	98	89	0.34	1.04	1.20	1.09	2N	NHMWM	n.d.	p.Arg854Gln	c.2561G > A	hom	Normal		**2N**
117	**57**	61	27	12	22	24	2.26	0.44	0.81	0.89	2M-GPIb	NHMWM	NHMWM	p.Pro293Glnfs*164 §	c.878delC §	het	Normal		**1**
118	**58**	53	31	25	30	41	1.71	0.81	0.97	1.32	1	NHMWM	NHMWM	p.Tyr897Serfs*12 §	c.2686delA §	het	Normal		**1**
	**59**	64	51	36	45	42	1.25	0.71	0.88	0.82	1	NHMWM	NHMWM	p.Tyr897Serfs*12 §	c.2686delA §	het	Normal		**1**
	**60**	76	36	36	41	52	2.11	1.00	1.14	1.44	1	NHMWM	n.d.	p.Tyr897Serfs*12 §	c.2686delA §	het	Normal		**1**
119	**61**	80	33	27	24	50	2.42	0.82	0.73	1.52	1	NHMWM	LHMWM	p.Arg924Gln<br>p.Cys2085Tyr §	c.2771G > A<br>c.6254G > A §	het het	Normal		**1**
	**62**	60	24	21	19	51	2.50	0.88	0.79	2.13	1	NHMWM	NHMWM	p.Cys2085Tyr §	c.6254G > A §	het	Normal		**1**
120	**63**	71	30	22	30	44	2.37	0.73	1.00	1.47	1	NHMWM	NHMWM	p.Pro812Argfs*31	c.2435delC	het	Normal		**1**
	**64**	109	59	63	54	52	1.85	1.07	0.92	0.88	1	NHMWM	n.d.	p.Pro812Argfs*31	c.2435delC	het	Normal		**1**
121	**65**	84	69	38	75	115	1.22	0.55	1.09	1.67	2M-GPIb	NHMWM	LHMWM	p.Ile1416Thr §	c.4247T > C §	het	Normal		**2M-GPIb**
	**66**	96	68	21	75	103	1.41	0.31	1.10	1.51	2M-GPIb	NHMWM	NHMWM	p.Ile1416Thr §	c.4247T > C §	het	Normal		**2M-GPIb**
122	**67**	65	55	46	59	73	1.18	0.84	1.07	1.33	1	NHMWM	NHMWM	p.Tyr1584Cys	c.4751A > G	het	Normal		**1**
	**68**	77	77	54	63	89	1.00	0.70	0.82	1.16	1	NHMWM	NHMWM	p.Tyr1584Cys	c.4751A > G	het	Normal		**1**
123	**69**	102	36	35	42	42	2.83	0.97	1.17	1.17	1	NHMWM	n.d.	No causative mutation found			Normal		**1**
	**70**	72	40	33	48	58	1.80	0.83	1.20	1.45	1	NHMWM	NHMWM	No causative mutation found			Normal		**1**
	**71**	40	25	28	28	50	1.60	1.12	1.12	2.00	1	NHMWM	n.d.	No causative mutation found			Normal		**1**
124	**72**	29	97	140	76	81	0.30	1.44	0.78	0.84	2N	NHMWM	NHMWM	p.Arg854Gln	c.2561G > A	hom	Normal		**2N**

 © 2022. The Author(s).

Supplementary Table S1 (*Continued*)

FN	UPN	FVIII	VWF: Ag	VWF: GPIb	VWF: CB	VWFpp	FVIII:C/ VWF:Ag	VWF: GPIb/ VWF:Ag	VWF:CB/ VWF:Ag	VWFpp/ VWF:Ag	Laboratory phenotype	VWF:MM, in-house	VWF:MM, Hydragel	HGVSp	HGVSc	Genotype	MLPA large deletion	Genotype	VWD type
125	**73**	13	29	22	29	55	0.45	0.76	1.00	1.90	2N	NHMWM	NHMWM	p.Pro812Argfs*31<br>p.Arg854Gln	c.2435delC<br>c.2561G > A	het het	Normal		**2N**
126	**74**	50	33	28	39	44	1.52	0.85	1.18	1.33	1	NHMWM	NHMWM	No causative mutation found			Exon 1–52	het	**1**
127	**75**	76	24	22	24	22	3.17	0.92	1.00	0.92	1	NHMWM	n.d.	p.Pro812Argfs*31	c.2435delC	het	Normal		**1**
	**76**	103	37	28	25	28	2.78	0.76	0.68	0.76	1	NHMWM	NHMWM	p.Pro812Argfs*31	c.2435delC	het	Normal		**1**
	**77**	155	86	74	75	63	1.80	0.86	0.87	0.73	1	NHMWM	NHMWM	p.Pro812ArgfsX31	c.2435delC	het	Normal		**1**
128	**78**	59	47	7	12	121	1.26	0.15	0.26	2.57	2A/B	LHMWM	LHMWM	p.Arg854Gln<br>p.Val1316Met	c.2561G > A<br>c.3946G > A	het het	Normal		**2B**
129	**79**	36	23	23	16	91	1.57	1.00	0.70	3.96	1	NHMWM	NHMWM	No causative mutation found			Normal		**1**
130	**80**	35	49	45	49	127	0.71	0.92	1.00	2.59	1	NHMWM	n.d.	p.Gly1579Arg	c.4735G > A	het	Normal		**2A/IIA**
132	**81**	26	19	26	12	135	1.37	1.37	0.63	7.11	1	NHMWM	NHMWM	p.Gly1775Asp §<br>p.Ala2569Ser §	c.5324G > A §<br>c.7706G > T §	het het	Normal		**1**
133	**82**	131	55	43	41	90	2.38	0.78	0.75	1.64	1	NHMWM	NHMWM	p.Glu2698Gly §	c.8093A > G §	het	Normal		**1**
134	**83**	78	47	30	33	189	1.66	0.64	0.70	4.02	1	LHMWM	LHMWM	p.Tyr1146Cys	c.3437A > G	het	Normal		**2A/IIE**
	**84**	39	24	15	13	79	1.63	0.63	0.54	3.29	2M-CB	LHMWM	LHMWM	p.Tyr1146Cys	c.3437A > G	het	Normal		**2A/IIE**
135	**85**	22	69	73	90	74	0.32	1.06	1.30	1.07	2N	NHMWM	n.d.	p.Arg854Trp<br>p.Arg854Gln	c.2560C > T<br>c.2561G > A	het het	Normal		**2N**
136	**86**	38	29	35	41	77	1.31	1.21	1.41	2.66	1	NHMWM	n.d.	p.Pro812Argfs*31	c.2435delC	het	Normal		**1**
	**87**	76	40	41	33	46	1.90	1.03	0.83	1.15	1	NHMWM	NHMWM	p.Pro812Argfs*31	c.2435delC	het	Normal		**1**
	**88**	74	38	42	36	47	1.95	1.11	0.95	1.24	1	NHMWM	n.d.	p.Pro812Argfs*31	c.2435delC	het	Normal		**1**
137	**89**	34	35	30	43	36	0.97	0.86	1.23	1.03	1	NHMWM	NHMWM	p.Asn166Ile	c.497A > T	het	Normal		**1**
	**90**	60	25	26	22	37	2.40	1.04	0.88	1.48	1	NHMWM	NHMWM	p.Asn166Ile	c.497A > T	het	Normal		**1**
	**91**	123	48	39	37	42	2.56	0.81	0.77	0.88	1	NHMWM	NHMWM	p.Asn166Ile	c.497A > T	het	Normal		**1**
	**92**	100	34	34	35	39	2.94	1.00	1.03	1.15	1	NHMWM	n.d.	p.Asn166Ile	c.497A > T	het	Normal		**1**
138	**93**	46	50	23	11	106	0.92	0.46	0.22	2.12	2A	AHMWM + LIMWM	AHMWM + LIMWM	p.Gly1609Arg	c.4825G > A	het	Normal		**2A/IIA**
	**94**	38	36	16	7	88	1.06	0.44	0.19	2.44	2A	AHMWM + LIMWM	AHMWM + LIMWM	p.Gly1609Arg	c.4825G > A	het	Normal		**2A/IIA**
139	**95**	56	31	28	18	98	1.81	0.90	0.58	3.16	2M-CB	LHMWM	LHMWM	p.Trp1144Gly	c.3430T > G	het	Normal		**2A/IIE**
140	**96**	20	96	79	62	106	0.21	0.82	0.65	1.10	2N	NHMWM	NHMWM	p.Arg854Gln	c.2561G > A	hom	Normal		**2N**
	**97**	159	65	73	59	92	2.45	1.12	0.91	1.42	1	NHMWM	n.d.	p.Arg854Gln	c.2561G > A	het	Normal		**1**
141	**98**	85	88	31	92	124	0.97	0.35	1.05	1.41	2M-GPIb	NHMWM	NHMWM	p.Glu1359Lys	c.4075G > A	het	Normal		**2M-GPIb**
	**99**	91	96	30	79	118	0.95	0.31	0.82	1.23	2M-GPIb	NHMWM	NHMWM	p.Glu1359Lys	c.4075G > A	het	Normal		**2M-GPIb**
142	**100**	50	18	20	19	36	2.78	1.11	1.06	2.00	1	NHMWM	n.d.	p.Gly160Trp	c.478G > T	het	Normal		**1**
	**101**	37	54	38	33	59	0.69	0.70	0.61	1.09	1	NHMWM	NHMWM	p.Gly160Trp	c.478G > T	het	Normal		**1**
143	**102**	28	23	21	18	126	1.22	0.91	0.78	5.48	1	NHMWM	NHMWM	p.Cys1165Trp	c.3495C > T	het	Normal		**1**
144	**103**	3	0	0	12	4					3	absent	n.d.	p.Pro812Argfs*31	c.2435delC	het	Normal		**3**
	**104**	134	66	62	56	63	2.03	0.94	0.85	0.95	1	NHMWM	NHMWM	p.Pro812Argfs*31	c.2435delC	het	Normal		**1**
145	**105**	111	34	32	26	55	3.26	0.94	0.76	1.62	1	NHMWM	NHMWM	p.Gln218His §	c.654G > T §	het	Normal		**1**
146	**106**	48	31	30	31	75	1.55	0.97	1.00	2.42	1	NHMWM	n.d.	p.Pro1266Leu p.<br>Val1279Ile	c.3797C > T<br>c.3835G > A	het het	Normal		**1**

(*Continued*)

 © 2022. The Author(s).

Supplementary Table S1 (*Continued*)

FN	UPN	FVIII	VWF: Ag	VWF: GPIb	VWF: CB	VWFpp	FVIII:C/ VWF:Ag	VWF: GPIb/ VWF:Ag	VWF:CB/ VWF:Ag	VWFpp/ VWF:Ag	Laboratory phenotype	VWF:MM, in-house	VWF:MM, Hydragel	HGVSp	HGVSc	Genotype	MLPA large deletion	Genotype	VWD type
147	**107**	41	24	12	6	77	1.71	0.50	0.25	3.21	2N/2M	AHMWM + LIMWM	AHMWM + LIMWM	p.Ser1506Leu	c.4571C > T	het	Normal		**2A/IIA**
148	**108**	69	91	33	80	93	0.76	0.36	0.88	1.02	2M-GPIb	NHMWM	NHMWM	p.Glu1359Lys p. pro2063Ser	c.4075G > A c.6187C > T	het het	Normal		**2M-GPIb**
	**109**	122	76	77	75	87	1.61	1.01	0.99	1.14	1	NHMWM	n.d.	p.Pro2063ser	c.6187C > T	het	Normal		**2M-GPIb**
150	**110**	134	82	11	28	156	1.63	0.13	0.34	1.90	2A	LHMWM	LHMWM	p.Ser1310Phe	c.3929C > T	het	Normal		**2B**
151	**111**	89	27	28	35	27	3.30	1.04	1.30	1.00	1	NHMWM	n.d.	p.Asn166Ile	c.497A > T	het	Normal		**1**
152	**112**	3	0	0	0	0					3	absent	n.d.	p.Asn166Ile p. Trp1025Glyfs*3	c.497A > T c.3072delC	het het	Normal		**3**
153	**113**	51	28	4	20	137	1.82	0.14	0.71	4.89	2M-GPIb	NHMWM	NHMWM	p.Pro1266Leu p. Val1279Ile	c.3797C > T c.3835G > A	het het	Normal		**1**
	**114**	94	84	105	117	120	1.12	1.25	1.39	1.43	1	NHMWM	n.d.	p.Pro1266Leu p. Val1279Ile	c.3797C > T c.3835G > A	het het	Normal		**1**
154	**115**	3	0	0	0	0					3	absent	n.d.	No causative mutation found			Exon 1–3	hom	**3**
155	**116**	91	58	57	61	57	1.57	0.98	1.05	0.98	1	NHMWM	n.d.	p.Pro812Argfs*31	c.2435delC	het	Normal		**1**
	**117**	3	0	0	0	2					3	absent	n.d.	p.Pro812Argfs*31	c.2435delC	hom	Normal		**3**
156	**118**	25	14	13	18	34	1.79	0.93	1.29	2.43	1	LHMWM	n.d.	p.Trp1144Gly	c.3430T > G	het	Normal		**2A/IIE**
	**119**	57	39	28	33	72	1.46	0.72	0.85	1.85	1	LHMWM	LHMWM	p.Trp1144Gly	c.3430T > G	het	Normal		**2A/IIE**
157	**120**	2	0	0	0	0					3	absent	n.d.	p.Arg1853*	c.5557C > T c.7730–1G > T	het het	Normal		**3**
158	**121**	3	0	0	0	3					3	absent	n.d.	p.Pro812Argfs*31	c.2435delC	hom	Normal		**3**
159	**122**	57	56	4	4	117	1.02	0.07	0.07	2.09	2A	AHMWM + LIMWM	AHMWM + LIMWM	p.Ile1628Thr	c.4883T > C	het	Normal		**2A/IIA**
	**123**	33	27	4	3	71	1.22	0.15	0.09	2.63	2A	AHMWM + LIMWM	AHMWM + LIMWM	p.Ile1628Thr	c.4883T > C	het	Normal		**2A/IIA**
	**124**	31	27	4	4	52	1.15	0.15	0.14	1.93	2A	AHMWM + LIMWM	AHMWM + LIMWM	p.Ile1628Thr	c.4883T > C	het	Normal		**2A/IIA**
160	**125**	92	80	85	111	71	1.15	1.06	1.39	0.89	1	NHMWM	n.d.		c.7730–1G > T	het	Normal		**1**
161	**126**	14	11	7	10	61	1.27	0.64	0.86	5.55	1	LHMWM	NHMWM	p.Trp1144Gly	c.3430T > G	het	Normal		**2A/IIE**
162	**127**	23	11	12	5	8	2.09	1.09	0.45	0.73	2M-CB	LHMWM	LHMWM	p.Pro812Argfs*31	c.2435delC	het	Normal		**1**
163	**128**	30	11	8	15	48	2.73	0.73	1.36	4.36	1	NHMWM	LHMWM	p.Val1414Glu §	c.2441T > A	het	Normal		**2M-GPIb**
	**129**	37	30	12	18	86	1.23	0.40	0.60	2.87	2M-GPIb	NHMWM	LHMWM	p.Val1414Glu §	c.2441T > A	het	Normal		**2M-GPIb**
164	**130**	62	61	60	61	89	1.02	0.98	1.00	1.46	1	NHMWM	n.d.	p.Pro1266Leu p. Val1279Ile	c.3797C > T c.3835G > A	het het	Normal		**1**
165	**131**	24	10	9	10	27	2.40	0.90	1.00	2.70	1	LHMWM	n.d.	p.Cys1196Tyr §	c.3585G > A	het	Normal		**2A/IIE**
166	**132**	7	0	0	0	4					3	absent	n.d.	No causative mutation found			Exon 1–3	hom	**3**
	**133**	70	41	52	41	51	1.71	1.27	1.00	1.24	1	NHMWM	n.d.	No causative mutation found			Exon 1–3	het	**1**
	**134**	85	34	35	40	41	2.50	1.03	1.18	1.21	1	NHMWM	n.d.	No causative mutation found			Exon 1–3	het	**1**
167	**135**	2	0	0	0	0					3	absent	n.d.	p.Ser85Pro p. Pro812Argfs*31	c.253T > C c.2435delC	het het	Exon 1–3	het	**3**
168	**136**	6	0	0	0	0					3	absent	n.d.	No causative mutation found			Exon 1–3	hom	**3**
169	**137**	48	49	49	49	76	0.98	1.00	1.01	1.55	1	NHMWM	n.d.	No causative mutation found			Normal		**1**
170	**138**	6	0	0	0	2					3	absent	n.d.	No causative mutation found			Exon 1–3	hom	**3**
	**139**	71	55	57	46	64	1.29	1.04	0.84	1.16	1	NHMWM	NHMWM	No causative mutation found			Exon 1–3	het	**1**
	**140**	3	0	0	0	0					3	absent	n.d.	No causative mutation found			Exon 1–3	hom	**3**
	**141**	112	29	33	32	30	3.86	1.14	1.10	1.03	1	NHMWM	n.d.	No causative mutation found			Exon 1–3	het	**1**
	**142**	144	57	56	57	42	2.53	0.98	1.00	0.74	1	NHMWM	n.d.	No causative mutation found			Exon 1–3	het	**1**

 © 2022. The Author(s).

Supplementary Table S1 (*Continued*)

FN	UPN	FVIII	VWF: Ag	VWF: GPIb	VWF: CB	VWFpp	FVIII:C/ VWF:Ag	VWF: GPIb/ VWF:Ag	VWF:CB/ VWF:Ag	VWFpp/ VWF:Ag	Laboratory phenotype	VWF:MM, in-house	VWF:MM, Hydragel	HGVSp	HGVSc	Genotype	MLPA large deletion	Genotype	VWD type
171	**143**	90	51	49	48	82	1.76	0.96	0.94	1.61	1	NHMWM	n.d.	No causative mutation found			Normal		**1**
	**144**	88	50	47	49	86	1.76	0.94	0.98	1.72	1	NHMWM	n.d.	No causative mutation found			Normal		**1**
	**145**	86	94	77	120	128	0.91	0.82	1.28	1.36	1	NHMWM	NHMWM	No causative mutation found			Normal		**1**
172	**146**	22	40	47	37	55	0.55	1.18	0.93	1.38	2N	NHMWM	n.d.	No causative mutation found			Normal		**1**
173	**147**	54	64	6	8	122	0.84	0.09	0.13	1.91	2A/B	LHMWM	LHMWM	p.Val1316Met	c.3946G > A	het	Normal		**2B**
	**148**	49	81	16	27	122	0.60	0.20	0.33	1.51	2A/B	LHMWM	LHMWM	p.Val1316Met	c.3946G > A	het	Normal		**2B**
	**149**	50	57	5	8	97	0.88	0.09	0.14	1.70	2A/B	LHMWM	LHMWM	p.Val1316Met	c.3946G > A	het	Normal		**2B**
174	**150**	0	0	0	0	0		0.00		0.00	3	absent	n.d.	p.Asn2636Thrfs*18 §	c.7907delA	het	Exon 1–3	het	**3**
175	**151**	116	57	58	34	63	2.04	1.02	0.60	1.11	1	NHMWM	NHMWM	No causative mutation found			Exon 1–3	het	**1**
176	**152**	30	17	16	24	103	1.76	0.94	1.41	6.06	1	LHMWM	n.d.	p.Trp1144Gly	c.3430T > G	het	Normal		**2A/IIE**
	**153**	4	0	0	0	13					3	absent	n.d.	p.Trp1144Gly	c.3430T > G c.6258 + 1dupT §	het het	Normal		**3**
177	**154**	39	40	14	13	98	0.98	0.35	0.33	2.45	2A	AHMWM + LIMWM	LHMWM	p.Arg1597Trp	c.4789C > T	het	Normal		**2A/IIA**
178	**155**	105	35	38	30	56	3.00	1.09	0.86	1.60	1	NHMWM	NHMWM	p.Pro812Argfs*31	c.2435delC	het	Normal		**1**
	**156**	91	54	21	26	3	1.69	0.39	0.48	0.06	2A	LHMWM	LHMWM	p.Pro812Argfs*31	c.2435delC	het	Normal		**1**
179	**157**	21	15	5	2	50	1.40	0.33	0.16	3.33	2A	AHMWM + LIMWM	AHMWM + LIMWM	p.Ser1506Leu	c.4517C > T	het	Normal		**2A/IIA**
180	**158**	3	0	0	0	4					3	absent	n.d.	p.Arg373*	c.1117C > T	hom	Exon 10	het	**3**
	**159**	2	0	0	0	3					3	absent	n.d.	p.Arg373*	c.1117C > T	hom	Exon 10	het	**3**
181	**160**	6	66	59	68	88	0.09	0.89	1.03	1.33	2N	NHMWM	NHMWM	p.Pro2063Ser p. Arg2342Cys §	c.6187C > T c.7024C > T	het het	Normal		**1**
182	**161**	8	0	0	0	2					3	absent	n.d.	p.Arg924Gln	c.2771G > A c.3379 + 1G > A	het het	Exon 1–3	het	**3**
183	**162**	22	11	9	13	56	2.00	0.82	1.18	5.09	1	NHMWM	LHMWM	p.Arg1315Cys	c.3943C > T	het	Normal		**2M-GPIb**
	**163**	33	13	10	14	53	2.54	0.77	1.08	4.08	1	NHMWM	LHMWM	p.Arg1315Cys	c.3943C > T	het	Normal		**2M-GPIb**
184	**164**	88	58	9	29	134	1.52	0.16	0.50	2.31	2A	LHMWM	LHMWM	p.Arg1374His	c.4121G > A	het	Normal		**2A-U**
	**165**	69	53	5	22	146	1.30	0.09	0.42	2.75	2A	LHMWM	LHMWM	p.Arg1374His	c.4121G > A	het	Normal		**2A-U**
	**166**	42	39	4	21	124	1.08	0.10	0.53	3.18	2A	LHMWM	LHMWM	p.Arg1374His	c.4121G > A	het	Normal		**2A-U**
185	**167**	107	43	46	52	65	2.49	1.07	1.21	1.51	1	NHMWM	n.d.	p.Arg1853*	c.5557C > T	het	Normal		**1**
	**168**	2	0	6	0	0					3	absent	n.d.	p.Q1556SfsX137 § p. Arg1853*	c.4666delC c.5557C > T	het het	Normal		**3**
	**169**	103	40	45	45	59	2.58	1.13	1.13	1.48	1	NHMWM	n.d.	p.Arg1853*	c.5557C > T	het	Normal		**1**
	**170**	144	65	71	89	57	2.22	1.09	1.37	0.88	1	NHMWM	n.d.	p.Q1556Sfs*137 §	c.4666delC	het	Normal		**1**
186	**171**	3	0	0	0	0					3	absent	n.d.	p.Pro812Argfs*31 p. Arg1853*	c.2435delC c.5557C > T	het het	Normal		**3**
187	**172**	51	21	21	12	1	2.43	1.00	0.55	0.05	2M-CB	LHMWM	LHMWM	no causative mutation found			Exon 1–3	hom	**1severe**
188	**173**	64	22	13	19	48	2.91	0.59	0.85	2.18	2M-GPIb	NHMWM	LHMWM	p.Arg1315Cys	c.3943C > T	het	Normal		**2M-GPIb**
189	**174**	69	52	55	62	81	1.33	1.06	1.19	1.56	1	NHMWM	n.d.	p.Asn1231Thr	c.3692A > C	het	Normal		**1**
190	**175**	96	59	67	70	64	1.63	1.14	1.19	1.08	1	NHMWM	n.d.	p.Pro1266Leu	c.3797C > T	het	Normal		**1**
191	**176**	40	46	65	55	90	0.87	1.41	1.20	1.96	1	NHMWM	n.d.	p.Arg1315Cys	c.3943C > T	het	Normal		**2M-GPIb**
192	**177**	26	10	5	10	41	2.60	0.50	0.96	4.10	2M-GPIb	NHMWM	LHMWM	p.Arg1315Cys	c.3943C > T	het	Normal		**2M-GPIb**
	**178**	45	24	16	16	47	1.88	0.67	0.68	1.96	1	NHMWM	LHMWM	p.Arg1315Cys	c.3943C > T	het	Normal		**2M-GPIb**

(*Continued*)

 © 2022. The Author(s).

Supplementary Table S1 (*Continued*)

FN	UPN	FVIII	VWF: Ag	VWF: GPIb	VWF: CB	VWFpp	FVIII:C/ VWF:Ag	VWF: GPIb/ VWF:Ag	VWF:CB/ VWF:Ag	VWFpp/ VWF:Ag	Laboratory phenotype	VWF:MM, in-house	VWF:MM, Hydragel	HGVSp	HGVSc	Genotype	MLPA large deletion	Genotype	VWD type
193	**179**	59	23	19	23	40	2.57	0.83	1.00	1.74	1	NHMWM	NHMWM	p.Ile1343Val p. Val1360Ala p. Phe1369Ile p. Ser1378Phe p. Arg1379Cys	c.4027A > G c.4079T > C c.4105T > A c.4133C > T c.4135C > T	het het	Normal		**1**
194	**180**	2	0	12	0	0		0.00		0.00	3	absent	n.d.	No causative mutation found			Exon 1–3	hom	**3**
195	**181**	30	17	13	15	34	1.76	0.76	0.88	2.00	1	NHMWM	LHMWM	p.Arg1315Cys	c.3943C > T	het	Normal		**2M-GPIb**
196	**182**	3	0	13	0	0		0.00		0.00	3	absent	n.d.	No causative mutation found			Normal		**3**
196	**183**	71	37	47	38	28	1.92	1.27	1.03	0.76	1	NHMWM	n.d.	No causative mutation found			Normal		**1**
197	**184**	38	16	23	17	62	2.38	1.44	1.06	3.88	1	NHMWM	n.d.	No causative mutation found			Normal		**1**
198	**185**	53	54	3	8	92	0.98	0.06	0.14	1.70	2A/B	AHMWM + LIMWM	AHMWM + LIMWM	p.Glu1638Lys	c.4912G > A	het	Normal		**2A/IIA**
198	**186**	37	32	3	8	61	1.16	0.09	0.24	1.91	2A/B	AHMWM + LIMWM	AHMWM + LIMWM	p.Glu1638Lys	c.4912G > A	het	Normal		**2A/IIA**
199	**187**	19	54	53	47	56	0.35	0.98	0.87	1.04	2N	NHMWM	NHMWM	p.Pro812Arg p. Arg854Gln	c.2435delC c.2561G > A	het het	Normal		**2N**
199	**188**	18	56	71	60	72	0.32	1.27	1.07	1.29	2N	NHMWM	NHMWM	p.Pro812Arg p. Arg854Gln	c.2435delC c.2561G > A	het het	Normal		**2N**
200	**189**	21	11	11	8	60	1.91	1.00	0.75	5.45	1	LHMWM	LHMWM	p.Trp1144Gly	c.3430T > G	het	Normal		**2A/IIE**
201	**190**	68	103	29	21	95	0.66	0.28	0.20	0.92	2A/B	LHMWM	LHMWM	p.Cys1190Phe §	c.3569G > T §	het	Normal		**2A/IIE**
202	**191**	28	8	4	4	1	3.50	0.50	0.55	0.13	1sev	NHMWM	LHMWM	p.Arg924Gln	c.2771G > A c.3379 + 1G > A	hom hom	Normal		**severe 1**

Abbreviations: Ag, antigen; AHMWM, absence of high-molecular-weight multimers; CB, collagen binding; FVIII, factor VIII; GPIb, glycoprotein Ib; het, heterozygous; hom, homozygous; H5/11VWM, Hydragel 5- or 11-VW multimer assay; LHMWM, loss of high-molecular-weight multimers; LIMWM, loss of intermediate-molecular-weight multimers; MLPA, multiplex ligation-dependent probe amplification; n.d., not determined; NHMWM, normal high-molecular-weight multimers; UPN, unique patient number; VWF, von Willebrand factor; VWFpp, von Willebrand factor propeptide; VWF:MM, von Willebrand factor multimeric analysis.

Note: §, mutations not previously described in the ISTH/EAHAD VWD database.

 © 2022. The Author(s).
